# Electroconvulsive therapy for manic state with mixed and psychotic features in a teenager with bipolar disorder and comorbid episodic obsessive–compulsive disorder: a case report

**DOI:** 10.1186/s13256-017-1508-8

**Published:** 2017-12-12

**Authors:** Olof Rask, Klara Suneson, Eva Holmström, Beata Bäckström, Björn Axel Johansson

**Affiliations:** 1 Department of Clinical Sciences, Division of Pediatrics, Lund University, Lund, Sweden; 2 Department of Clinical Sciences, Division of Psychiatry, Lund University, Lund, Sweden; 3 Department of Clinical Sciences Lund, Division of Child & Adolescent Psychiatry, Lund University, Lund, Sweden; 4 Office for Healthcare ‘Sund’, Child & Adolescent Psychiatry, Regional Inpatient Care, Emergency Unit, Malmö, Sweden; 5 Office for Healthcare ‘Sund’, Child & Adolescent Psychiatry, Unit for Pediatric Bipolar & Psychotic Disorders, Lund, Sweden

**Keywords:** Adolescents, Bipolar disorder, Comorbidity, *Diagnostic and Statistical Manual of Mental Disorders*, Electroconvulsive therapy, Obsessive-compulsive disorder

## Abstract

**Background:**

Comorbidity of bipolar disorder and obsessive–compulsive disorder is common in adolescence. Obsessive–compulsive disorder symptoms may be episodic and secondary to alterations in mood, and display specific features. Management of pediatric bipolar disorder-obsessive–compulsive disorder is challenging, as pharmacotherapy of obsessive–compulsive disorder may induce or exacerbate manic episodes and there is limited evidence of treatment efficacy. Electroconvulsive therapy is sparsely used in children and adolescents, but is documented to be a safe and efficacious intervention in adults with bipolar disorder. In view of the severity of symptoms in juvenile mania, studies on treatment strategies are warranted. We report a case of an adolescent with bipolar disorder-obsessive–compulsive disorder who was successfully treated with electroconvulsive therapy during an episode of severe mania.

**Case presentation:**

A 16-year-old girl of Middle East origin first presented to us with depressed mood, irritability, and increased obsessive–compulsive disorder symptoms, which were initially interpreted in the context of acute stress secondary to migration. She had been diagnosed with bipolar disorder and obsessive–compulsive disorder in her previous home country, but had difficulties in accounting for earlier psychiatric history. During hospitalization her mood switched to a manic state with mixed and psychotic features, at times showing aggression toward others. Interruption in her lithium treatment for a short period and possibly the introduction of an atypical antipsychotic could in part have been triggering factors. After 8 weeks of in-patient care and psychotropic drug trials, electroconvulsive therapy was initiated and administered every second or third day for 4 weeks, with marked positive response. No apparent side effects were reported.

**Conclusions:**

This case demonstrates the need for a detailed medical history, taking special note of periodicity and character of obsessive–compulsive disorder symptoms, in adolescents with mood disorders. When treating culturally diverse patients, extra consideration should be taken. Special concerns in the pharmacological treatment to avoid the patient’s condition from worsening must be addressed, including giving priority to mood stabilization before obsessive–compulsive disorder symptoms. There are potential benefits in considering electroconvulsive therapy in young patients with severe mania where first-line treatment options have failed.

## Background

Comorbidity of bipolar disorder (BD) and obsessive–compulsive disorder (OCD) is a common condition (BD-OCD) among adolescents. Previous pediatric studies have found that as many as 21% of patients with BD and 15% of patients with OCD meet *Diagnostic and Statistical Manual of Mental Disorders*, Third Edition, Revised (DSM-III-R) criteria for both disorders [1]. Clinical management is challenging as mood stabilizers, such as lithium, show marginal efficacy in treating OCD symptoms, and treatment of adolescents with clomipramine, selective serotonin reuptake inhibitors (SSRIs), and possibly also some second-generation neuroleptics could induce or exacerbate mood instability and precipitate mania [2–4]. There is limited evidence of treatment efficacy of commonly used drugs in pediatric patients with BD-OCD, who are often treated with polypharmacy [5]. Furthermore, psychotherapeutic approaches for treating OCD may be hampered by unstable BD.

Pediatric mania is heterogeneous in its presentation across individuals. Several concurrent symptoms, including mood lability (rapid mood variation in a short time) and increased energy and irritability are commonly present [6]. Adolescents with BD tend to show long episodes with mixed symptomatology; the mixed symptomatology is an overlapping of manic and depressive features, which may be difficult to treat and seem to be associated with high rates of relapse [7]. Physical aggression toward others is common, and has been reported to be more frequent when mania is accompanied by depressive symptoms; these episodes also appear in adults [8].

In the fifth edition of the *Diagnostic and Statistical Manual of Mental Disorders* (DSM-5), multiple specifiers, such as psychotic or mixed features, are included to describe the clinical course of a person’s affective episode [9]. The previous entity of a mixed episode of BD is thus replaced with the specifier “with mixed features,” which, however, does not include symptoms such as agitation and irritability. It is yet to be determined if this DSM-5 classification will identify new subgroups of patients with mixed symptomatology, and what therapeutic implications this could have.

Electroconvulsive therapy (ECT) is documented to be a safe and efficacious intervention in manic, depressed, and mixed states of BD and in a few cases of OCD in adults [10, 11]. ECT is rarely used in adolescents, usually only for indications that involve severe depression or life-threatening conditions, for example catatonia [12–15]. In view of the severity of juvenile mania, further studies are needed about treatment strategies, including ECT and its potential effects, in children and adolescents with BD-OCD. Well-tolerated interventions that can produce remission and promote positive functioning are much needed.

Here, we report a case of a teenage patient with BD-OCD who was successfully treated with ECT during an episode of severe mania with mixed and psychotic features. She first presented at our emergency unit with depressed mood and increased OCD symptoms, which initially were interpreted in the context of acute stress secondary to recent migration. This case demonstrates specific diagnostic and treatment challenges of which the physician managing adolescents with BD-OCD should be aware, as well as possible therapeutic strategies.

## Case presentation

### Previous history

Our patient, a 16-year-old girl of Middle East origin, arrived in Sweden with her family as a refugee in 2016. The family had lived under traumatic circumstances and had been forced to flee due to the political situation in their country of origin. On arrival in Sweden, our patient was under treatment with lithium, levothyroxine, and haloperidol, and was enrolled in our out-patient care unit for pediatric bipolar and psychotic disorders. Her parents showed a medical document saying she was diagnosed with BD and OCD by a psychiatrist in a major city in the Middle East. However, the parents and our patient had difficulties in accounting for her psychiatric history and showed little knowledge about the disorders and why she was taking the prescribed medicines.

She had no family history of psychiatric diseases. Her psychomotor development in childhood was reported normal. She had been physically active, successfully participated in martial arts, and was described as helpful, responsible, and having friends, but with no academic schooling. The first psychiatric symptom observed in our patient, according to her parents, was an episode of altered mood at 14 years of age. During this episode she was unable to get up from bed for 1 month, stopped eating and drinking, and needed help to visit the bathroom. She was prescribed psychopharmacological treatment and recovered after approximately 3 more weeks. This episode was followed by a more active episode with disruptive behavior, including self-harm by cutting her ankles. There were no reports of suicide attempts.

### Out-patient care

During her first months in Sweden, our patient’s main psychiatric problems were irritability, aggressiveness toward herself and her sister, mood shifts, and sleep difficulties. Her mood stabilized somewhat after an increased dose of lithium, even though she was under pressure from the process of seeking asylum and waiting for decisions from the authorities. She had great difficulties in recalling any previous episodes of mania or depression, but could describe periods of obsessive thoughts, mainly of sexual and religious character, since childhood. She felt shameful about these thoughts and about previous reckless behavior.

Additional treatment with quetiapine was initiated at a low dose of 25 mg daily. After a conflict with her parents, our patient stopped taking her medications, including lithium, for a few days. Shortly after this episode of altered compliance, she was exposed to several additional psychosocial stress factors. These included interviews at the migration office and deportation of an elder brother and his 4-year-old son, at the same time as she started school. Our patient presented severe anxiety, disturbing sinful thoughts, and depressive and suicidal ideations. She was subsequently admitted for emergency care at the Department of Child & Adolescent Psychiatry in Malmö, 7 months after arriving in Sweden.

### In-patient care, course of acute illness

During the intake interview, our patient expressed a strong sense of guilt and distress because of her obsessive thoughts, with fear that she might harm herself or others, show inappropriate sexual behavior, or offend religious objects and thereby dishonor her religion. At the ward, compulsive behavior was noted, including excessive hand washing, showering procedures, and a need to line up different items on her bed with great accuracy. She did not wish to continue treatment with lithium, in part because she experienced adverse effects such as tremor and because of the need for repeated blood sampling. Her serum lithium level was 1.3 mmol/L (0.5 to 1.2 mmol/L) 2 days after admission. Our patient’s negative attitude, possible side effects, and the clinical picture dominated by depressive mood and OCD symptomatology, led to the decision to phase out lithium. Over the following 2 weeks, lithium was removed from her treatment and simultaneously quetiapine was up-titrated to 600 mg daily.

Our patient underwent magnetic resonance imaging (MRI) of her brain that showed no abnormalities. Routine laboratory tests indicated mild under-treatment of her hypothyroidism and there were no suspicions of illicit drug use. The Children’s Yale-Brown Obsessive Compulsive Scale (CY-BOCS) showed a total score of 18 points, indicating moderate OCD severity.

During the third and fourth week of in-patient care, our patient experienced a certain degree of mood stabilization and less anxiety. However, during the fifth week, her mood switched to a manic state with mixed and psychotic features. Further care was given according to the Swedish Compulsory Mental Care Act. Our patient showed a complex symptomatology, with rapid switches from euphoria with elevated energy to episodes of dysphoria with depressed mood, feelings of worthlessness, and fatigue. Overlapping manic and depressive symptoms such as psychomotor agitation, irritability, and distractibility were present, as well as paranoid delusions such as fear of being poisoned. Her pattern of sleep was severely impaired with a decreased need for sleep. In her room, she tore away some of the base-boards, on one occasion using them as a weapon against the staff. At times she was also aggressive toward fellow patients, leading to recurrent periods of seclusion. At other times she planned a wedding to take place between herself and members of the staff. Occasionally, she sang and laughed inappropriately or excessively, at other moments she kicked and punched the walls. For a few days, she refused oral medication. In this severe state, forced injections with haloperidol, zuclopenthixol acetate, and diazepam respectively were needed on various occasions to prevent self-harm and injury to others.

Despite reinstatement of lithium and further elevation of quetiapine to 800 mg daily, our patient did not improve during the following 2 weeks. Blood samples showed serum lithium concentrations of 0.7 to 0.9 mmol/L. At the beginning of the eighth week, haloperidol, which had previously shown a stabilizing effect in our patient, was reinstated and increased to 4 mg daily, while quetiapine was successively lowered to 150 mg daily. However, our patient’s behavior was continuously labile, with episodes of hyperactivity, agitation, and delusions.

**Fig. 1:**
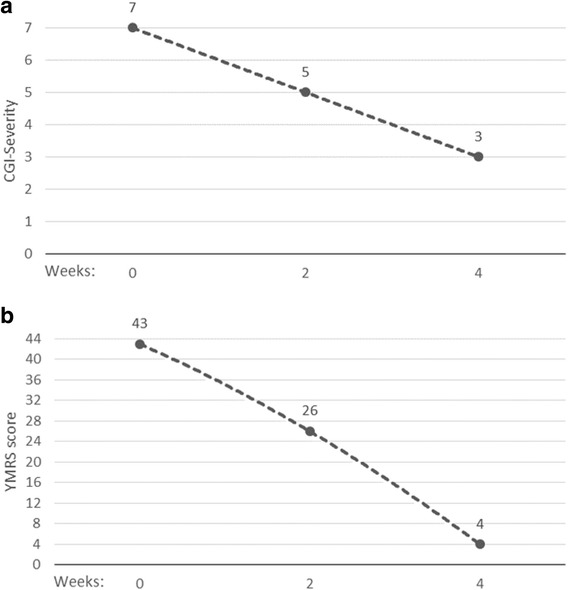
Severity of illness after start of electroconvulsive therapy, as described in Clinical Global Impression (**a**) and Ziegler Young Mania Rating Scale score (**b**) *CGI* Clinical Global Impression, *YMRS* Ziegler Young Mania Rating Scale

Clinical discussions ensued regarding further psychotropic drug trials versus ECT; ECT was initiated with a first administration during the ninth week of hospitalization. ECT was administered every second or third day for 4 weeks with a total of 11 treatments. Unilateral ECT according to d’Élia [16] was used with a dose of 96.0 to 156.8 millicoulombs (mC) and a pulse-width of 0.3 to 0.35 milliseconds (ms). The average seizure length was 23 seconds (range 16 to 37 seconds). Propofol was used as an anesthetic agent in ten sessions and thiopental was used in one session. Our patient showed a prompt and marked positive response to treatment, with a dramatic decrease in her severity of illness, as described by using the Ziegler Young Mania Rating Scale (YMRS) and Clinical Global Impression (CGI) scores (Fig. 1a and b). She experienced no evident negative side effects. Fourteen weeks after admission, our patient could be discharged to stay with her family. Her base medication at discharge was lithium, with a serum concentration of 0.7 mmol/L, haloperidol 4 mg daily, and quetiapine 150 mg daily, with a plan of further tapering of antipsychotics.

### Follow-up period

After discharge, our patient was reviewed by our specialized out-patient care unit and monitored by home visits twice a week. The lithium concentration was controlled once a week and kept at 0.9 to 1.1 mmol/L. Psychosocial interventions including psychoeducation about BD and OCD were conducted. Psychotic and OCD symptoms were meliorated and new mood episodes have not yet been observed during the current 3-month follow-up period. Our patient has restarted school and is actively taking part in some physical activities. The family is still waiting for final decisions about permanent residency in Sweden.

After discharge, we established contact (by telephone and email) with our patient’s physician in the family’s previous country. We were informed that our patient had been treated for approximately 2 years in their out-patient clinic, and that her OCD symptoms most probably were related to her BD, as decreased obsessions and compulsions had been noted when her mood was normalized. Our foreign colleague emphasized that there had often been a high level of expressed emotions within the family.

## Discussion and conclusions

We present this case as it highlights several possible diagnostic and clinical pitfalls in child and adolescent psychiatry, especially when comorbid symptoms are present, as well as therapeutic considerations, including ECT, which may be underused as anti-manic treatment among adolescents.

BD and OCD have been identified as a common comorbidity [1, 17]. This has raised a question of whether the comorbidity represents distinct clinical entities or multiple symptoms of one disease. It has been suggested that most comorbid OCD cases may in fact be secondary to mood episodes of BD, whereas some comorbid OCD cases are non-episodic and thus could represent “true” OCD independent of BD [17]. It has also been reported that the types of OCD symptoms in BD-OCD are somewhat different from pure OCD; obsessions found in BD-OCD tend to be more often of a sexual, aggressive, and religious nature [18], as was the case with our patient. Other authors have found that patients with BD-OCD, as compared to those without comorbidity, are younger at onset of mood episodes and more often have a history of previous suicidal attempts, seasonality, rapid cycling, and impulsivity [19]. This underlines the significance of evaluating young patients with BD or OCD for reciprocal comorbidity.

Our patient presented with a depressed mood and increased OCD symptoms in the context of several social stress factors, and switched into a severe manic mood episode with mixed and psychotic features during hospitalization. The reintroduction of a second-generation antipsychotic (quetiapine) with increased dosage as lithium was withdrawn for a short period after admission may have been a switch-triggering factor [20]. However, this was less likely in this case, as 3 months after discharge our patient is doing well with only mild OCD symptoms on lithium and quetiapine 450 mg daily. If a patient’s symptoms continue to worsen while taking a specific mood stabilizer, it is advisable to consider an alternative treatment strategy, for example ECT. In our patient, the lithium concentration could theoretically have been further elevated after her switch into her manic state. Aggravation of side effects, as in our patient, may sometimes hinder this and necessitate alternative strategies.

This case also demonstrates the need for a detailed medical history to promote clinical diagnostic reasoning and increase awareness of the treatment challenges of BD-OCD comorbidity. Acute psychotic symptoms with disorganized behavior could have been interpreted as a schizophreniform disorder. However, the longitudinal course of her illness with psychotic symptoms only during an acute manic episode makes this unlikely. Retrospectively, the specific character and episodic nature of our patient’s OCD symptoms in her previous history fits well with the special features of BD-OCD, as previously described. If we had tried more rigorously to access this information, in collaboration with our patient’s earlier physicians, we might have been more reluctant to adhere to our patient’s wish to discontinue lithium treatment at the time of admission. This case also underlines that psychiatric assessment requires extra considerations and efforts in patients who are culturally and linguistically diverse [21].

There is strong evidence supporting the efficacy and safety of ECT as an acute antidepressant in adolescent psychiatry; less commonly known is the effectiveness of ECT as a mood stabilizer and anti-manic treatment, and thereby also as possible treatment in bipolar states with mixed features [10, 11]. ECT seemingly continues to be at the very bottom of pediatric treatment algorithms [14, 22]. This may unnecessarily prolong treatment of severely ill children and adolescents. Our patient demonstrated a rapid clinical response to ECT, but it remains to be established whether ECT could be particularly effective for certain subgroups of patients with BD-OCD.

In conclusion, this case demonstrates several specific clinical features of pediatric BD-OCD, such as periodicity and the character of obsessions and mixed symptomatology; it is crucial for the treating physician to be aware of and to look for specific clinical features of pediatric BD-OCD. Diagnosis should routinely involve a collateral informant familiar with the child’s previous behavior, which may be especially important in migration medicine. Key points in the pharmacological treatment aimed at preventing worsening of the patient’s condition include giving priority to mood stabilization before OCD symptoms. There are potential benefits in considering ECT in adolescent patients with severe mania where first-line treatment options have failed; further clinical research on the use of ECT in pediatric patients would help advance current knowledge.

## Abbreviations

BD: Bipolar disorder
CGI: Clinical Global Impression
CY-BOCS: Children’s Yale-Brown Obsessive Compulsive Scale
DSM-III-R: *Diagnostic and Statistical Manual of Mental Disorders*, Third Edition, Revised
DSM-5: *Diagnostic and Statistical Manual of Mental Disorders*, Fifth Edition
ECT: Electroconvulsive therapy
MRI: Magnetic resonance imaging
OCD: Obsessive–compulsive disorder
SSRI: Selective serotonin reuptake inhibitor
YMRS: Young Mania Rating Scale
